# Piccolo Directs Activity Dependent F-Actin Assembly from Presynaptic Active Zones via Daam1

**DOI:** 10.1371/journal.pone.0120093

**Published:** 2015-04-21

**Authors:** Dhananjay Wagh, Ryan Terry-Lorenzo, Clarissa L. Waites, Sergio A. Leal-Ortiz, Christoph Maas, Richard J. Reimer, Craig C. Garner

**Affiliations:** 1 Department of Psychiatry and Behavioral Sciences, Nancy Pritzker Laboratory, Stanford University, Stanford, California, United States of America; 2 Department of Pathology and Cell Biology Columbia University New York, New York, United States of America; 3 Department of Neurology and Neurological Sciences Stanford University and Veterans Affairs Palo Alto Health Care System, Palo Alto, California, United States of America; University of Toronto, CANADA

## Abstract

The dynamic assembly of filamentous (F) actin plays essential roles in the assembly of presynaptic boutons, the fusion, mobilization and recycling of synaptic vesicles (SVs), and presynaptic forms of plasticity. However, the molecular mechanisms that regulate the temporal and spatial assembly of presynaptic F-actin remain largely unknown. Similar to other F-actin rich membrane specializations, presynaptic boutons contain a set of molecules that respond to cellular cues and trans-synaptic signals to facilitate activity-dependent assembly of F-actin. The presynaptic active zone (AZ) protein Piccolo has recently been identified as a key regulator of neurotransmitter release during SV cycling. It does so by coordinating the activity-dependent assembly of F-Actin and the dynamics of key plasticity molecules including Synapsin1, Profilin and CaMKII. The multidomain structure of Piccolo, its exquisite association with the AZ, and its ability to interact with a number of actin-associated proteins suggest that Piccolo may function as a platform to coordinate the spatial assembly of F-actin. Here we have identified Daam1, a Formin that functions with Profilin to drive F-actin assembly, as a novel Piccolo binding partner. We also found that within cells Daam1 activation promotes Piccolo binding, an interaction that can spatially direct the polymerization of F-Actin. Moreover, similar to Piccolo and Profilin, Daam1 loss of function impairs presynaptic-F-actin assembly in neurons. These data suggest a model in which Piccolo directs the assembly of presynaptic F-Actin from the AZ by scaffolding key actin regulatory proteins including Daam1.

## Introduction

Activity-dependent neurotransmitter release, involving the docking, priming and fusion of synaptic vesicles (SVs) with the AZ plasma membrane, is the central function of presynaptic terminals [1, 2]. Efficient neurotransmission, particularly during periods of sustained neuronal activity, also requires the recruitment of SVs from the reserve pool (RP) to the readily releasable pool (RRP), and the recycling of SV proteins following vesicle fusion [1, 3]. Although a growing number of molecules that mediate SV priming, fusion and recycling have been identified, our understanding of how SVs are maintained within boutons while readily transitioning between readily releasable, recycling, and reserve pools is less clear. Microfilaments represent a highly dynamic cytoskeletal system implicated in facilitating several of these transitions. For example, polymerized F-actin is critical for retaining SVs in the RP through their mutual interactions with Synapsin [4, 5] and facilitates SV translocation to the RRP through myosin motors [6–8]. F-actin has also been found to negatively regulate SV release probability (*P*_*vr*_) by creating a barrier to restrain SV fusion at the AZ [7, 9]. Microfilaments also contribute to SV endocytosis and recycling in conjunction with Dynamin, Abp1, and Synapsin [10–15] as well as the sharing of SVs between neighboring boutons [16]. Intriguingly, the dynamic assembly of presynaptic F-actin also participates in the functional plasticity of presynaptic boutons and cognitive function [7, 8, 17], through mechanisms that may involve the active recruitment of the plasticity molecule CaMKII [18].

At present, our understanding of the molecules that temporally and spatially regulate presynaptic F-actin assembly is limited. The few that have been identified include Profilin2, a globular (G)-actin binding ATP/ADP exchange factor found to regulate *P*_*vr*_ [17], and CDC42, a Rho family GTPase that can mediate the awakening of immature pre-synaptically silent synapses as part of the TrkB/BDNF signaling pathway [19]. In addition, the cell adhesion molecule N-Cadherin, which can trans-synaptically regulate synaptic plasticity [20] modulates presynaptic actin assembly [21] as does N-WASP which binds actin monomers and the Arp2/3 complex, which in turn creates new filaments as branches on older actin filaments [22]. RhoA, another Rho family GTPase has also been found to modulate presynaptic F-actin assembly [23].

Dynamic imaging and ultrastructural studies suggest that the presynaptic AZ and peri-active zone regions within nerve terminal represent nucleation sites for the activity dependent assembly of F-actin [7, 9, 13, 24–27]. While the periactive zone regions within nerve terminal have been coupled to endocytosis and the nucleation of F-actin by the endocytic machinery [10–15, 26], how the AZ might regulate the focal assembly of F-actin during the translocation, fusion and recycling of SVs is unclear.

Studies focused on elucidating the functions of the presynaptic AZ protein Piccolo suggest that this multidomain scaffold molecule is a key regulator of AZ mediated F-actin assembly [18]. Piccolo has been shown to interact with a number of Actin regulatory proteins including Abp1 [12], GIT1 [28], Profilin2 [18, 29], and cAMP-GEF II/Epac2 [30]. In addition, stabilizing F-actin with Jasplakinolide reverses the enhanced rates of activity-dependent SV exocytosis and Synapsin1a dispersion associated with loss of Piccolo expression [31]. This suggests that Piccolo executes its effects on SV exocytosis, in part, by regulating the dynamic assembly of F-actin [18]. This concept is further supported by experiments demonstrating that activity dependent assembly of presynaptic F-actin is abolished in the absence of Piccolo or its binding partner Profilin2 [18].

In the present study, we have identified Daam1 as a novel Piccolo binding partner. Daam1 belongs to the family of Diaphanous-Related Formins (DRFs) [32, 33] that operates in conjunction with Profilin [34] to catalyze the polymerization of Actin following its activation by the Wnt/Disheveled signaling complex [32, 35]. Our studies reveal that Daam1 physically interacts with Piccolo and is present at synapses. Moreover, similar to loss of Piccolo and Profilin2, Daam1 loss of function impairs presynaptic F-actin assembly, suggesting that within AZs Piccolo/Profilin/Daam1 could serve as a regulated nucleation site for F-actin assembly.

## Results

### Daam1 co-localizes with Piccolo within presynaptic boutons

**Fig 1:**
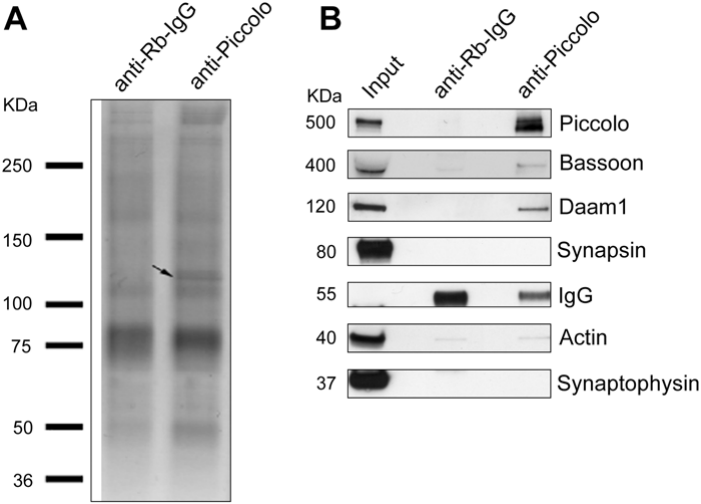
Daam1 is a novel binding partner of Piccolo. (***A*)** Silver stained SDS PAGE gel of Piccolo antibody immunoprecipitation from P4 rat brain light membrane fraction reveals the presence of a 120 KDa band that was identified as Daam1 by mass spectrometry. **(*B*)** Western Blot analyses of similar SDS PAGE gels confirm the presence Daam1 in Piccolo immunoprecipitated fractions. Note that Bassoon, a known binding partner of Piccolo, is also immunoprecipitated while two other presynaptic proteins, Synapsin and Synaptophysin are not.

To understand better the physiological role of Piccolo we sought to identify its binding partners by immunoprecipitating Piccolo from the light membrane fraction of brains from post-natal day 4 (P4) rats using an affinity purified Piccolo polyclonal antibody directed against the Piccolo residues 1980–2553 (Pclo_1980-2553_). Silver staining of immunoprecipitated proteins separated by SDS-PAGE revealed the presence of a prominent band at 120 kDa which was not present in IgG control precipitates (Fig 1A). Mass-spectrometric analysis of this band demonstrated that it contains the protein Daam1. Western blot analysis with Daam1 specific antibodies confirmed its presence in the Piccolo antibody immunoprecipitate from brain lysates (Fig 1B). Western blot analysis also identified Bassoon, a known binding partner of Piccolo [36], in the immunoprecipitate, but not Actin or the SV-associated proteins Synaptophysin and Synapsin, supporting the specificity of the immunoprecipitation (Fig 1B).

**Fig 2:**
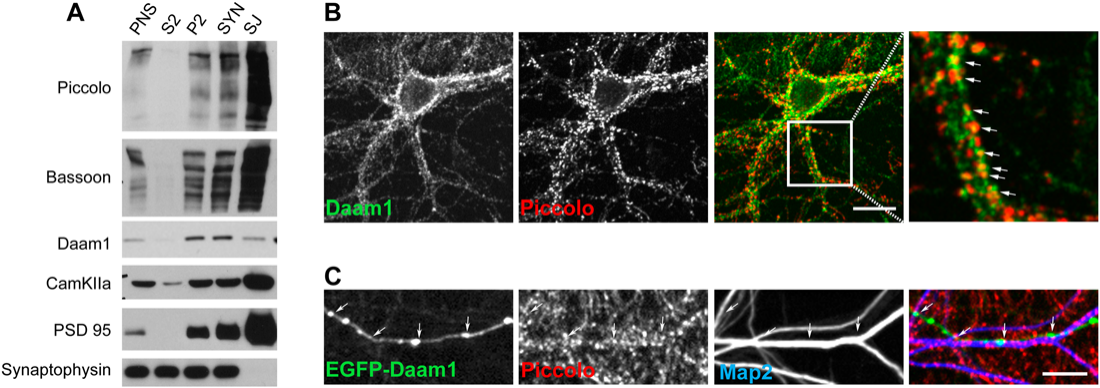
Daam1 is present within the presynaptic bouton. **(*A*)** Western blot analysis of fractions generated by differential centrifugation of adult brain lysate demonstrates the presence of Daam1 in synaptosomes (SYN) and to a lesser extent synaptic junctions (SJ). Signals with antibodies to known synaptic junctional components (Bassoon, Piccolo, CaMKII and PSD-95) versus a synaptic vesicle protein (Synaptophysin) demonstrate the integrity of the preparation. PNS—Post nuclear supernatant, S2- post hypotonic lysis supernatant, P2- post-hypotonic lysis pellet, SYN—synaptosomes, SJ—synaptic junctions. **(*B*)** Immmunostaining of cultured hippocampal neurons (16 DIV) with antibodies against Daam1 (green) and Piccolo (red) reveals a synaptic colocalization of the two endogenous proteins. Scale bar is 5 μm. **(*C*)** Axons from EGFP-Daam1 (green) expressing neuron (14 DIV, transfected at DIV 0) immunostained with antibodies against Piccolo (red) and MAP2 (blue) reveal EGFP-Daam1 puncta within Piccolo positive presynaptic boutons juxtaposed to MAP2 positive dendrites. Scale bar is 5 μm.

Daam1 is a processive Formin that has been shown to polymerize Actin monomers into F-actin both *in vivo* and *in vitro* [35, 37]. It has also been linked to the growth of presynaptic boutons at the *Drosophila* neuromuscular junction [38]. This observation and the biochemical interaction with Piccolo suggest that Daam1 should be present within presynaptic boutons. Similar to previous studies [39], we found that Daam1 was enriched in synaptosomes and to a lesser degree with purified synaptic junction preparations isolated by differential centrifugation (Fig 2A), indicating that it is present in synaptic membranes, but is more loosely associated with synapses than Piccolo, Bassoon, CaMKII, and PSD-95. We also immunostained cultures of dissociated hippocampal neuronal grown for 16 days *in vitro* (16 DIV) and found Daam1 immunoreactivity exhibited a punctate expression pattern along soma and dendritic arbors of hippocampal pyramidal cells that colocalized with Piccolo positive puncta (Fig 2B). In transfected cells, recombinant EGFP-tagged Daam1 colocalized with Piccolo positive axonal varicosities that abutted MAP2 stained dendrites consistent a presynaptic localization of Daam1 (Fig 2C). The EGFP-tagged Daam1 also localized to dendritic spines (data not shown).

### Daam1 interacts with the central region of Piccolo

**Fig 3:**
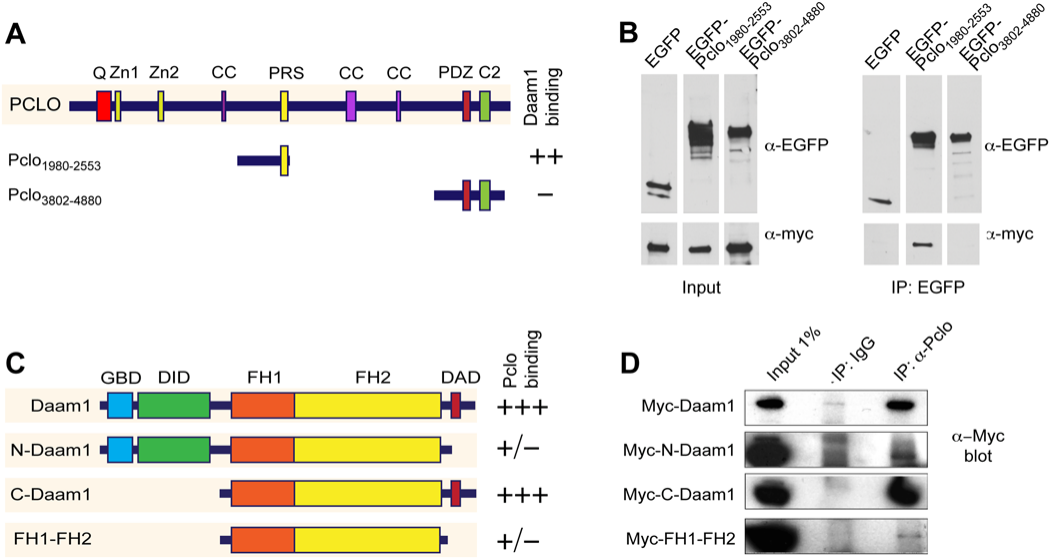
Daam1 interacts with the central region of Piccolo. (***A***) Schematic diagram of Piccolo depicting relative positions of different subdomains and composition of EGFP-tagged cDNA clones used in pull-down assays. **(*B*)** Western blot analysis of EGFP-tagged Piccolo fragments and Myc-Daam1 expressed and immunoprecipitated from COS7 cells with an antibody to GFP. (***C***) Schematic diagram of Daam1 depicting relative positions of different subdomains and organization of Myc-tagged cDNA clones. **(*D*)** Western blot analysis of Myc-tagged Daam1 constructs co-expressed with EGFP-Pclo_1980-2553_ and immunoprecipitated from COS7 cells with an antibody to Piccolo. Q—polyQ domain; Zn1 and Zn2—zinc fingers 1 and 2; CC—coiled coil domain; PRS—proline rich sequence; PDZ—PDZ domain; C2—C2 domain; GBD—Rho GTPase binding domain; DID—Diaphanous Inhibitory Domain; FH1 and FH2—formin homology domains 1 and 2; DAD—Diaphanous Autoregulatory Domain.

Coimmunoprecipitation of Daam1 with Piccolo from rat brain lysates indicates that the two proteins may interact directly. Unexpectedly, we found that a second polyclonal antibody also directed against Pclo_1980-2553_ was unable to co-immunoprecipitate Daam1 (data not shown), suggesting that Daam1, binding near or at this site, might cause epitope masking. We therefore examined whether recombinant Daam1 would form a complex with Pclo_1980-2553_ when co-transfected into COS7 cells (Fig 3A and 3B). As predicted, we were able to co-precipitate Myc- tagged Daam1 with an anti-GFP antibody when it was expressed with EGFP-Pclo_1980-2553_, but not EGFP-tagged C-terminal segment of Piccolo (EGFP-Pclo_3802-4880_). Interestingly, Pclo_1980-2553_ is not conserved in the closely related Bassoon [40], but interacts with other Actin-associated proteins including GIT1 and Profilin1 and Profilin2 suggesting that this region of Piccolo may have a unique role in presynaptic F-actin assembly [18, 28, 31].

We next utilized an analogous approach to identify the region in Daam1 that interacts with Pclo_1980-2553_ (Fig 3C and 3D). As expected, EGFP antibodies coimmunoprecipitated full-length Daam1 (Myc-Daam1) when expressed with EGFP-Pclo_1980-2553_. We similarly found that an isoform of Daam1 that lacks the N-terminus including the RhoA GTPase Binding Domain (GBD) and Diaphonous Inhibitory Domain (DID) of the protein (Myc-C-Daam1; residues 428–1076) coimmunoprecipitated with EGFP-Pclo_1980-2553_. However, two truncated variants of Daam1, Myc-N-Daam1 (residues 1–986) and the FH1-FH2 fragment of Daam1 (residues 428–986) were not effectively precipitated with EGFP-Pclo_1980-2553_. Both of these isoforms lack the C-terminus including the Diaphanous Autoregulatory Domain (DAD) of the protein, suggesting that Daam1-Piccolo interaction may require residues within this region of Daam1.

### Functional relevance of the Daam1-Pclo_1980-2553_ interaction

In addition to interacting with Daam1, Pclo_1980-2553_ contains a proline rich region that binds the G-actin ADP/ATP exchange protein Profilin [29], which in conjunction with Daam1 promotes F-actin assembly [35, 41, 42]. Pclo_1980-2553_ also contains a 153 amino acid fragment (residues 2197–2350) that binds GIT1, a multidomain GTPase activating protein (GAP) for ADP-ribosylation factors (ARFs) [28]. GIT1 has additional binding sites for proteins involved in focal adhesions including βPIX, Focal Adhesion Kinase (FAK), and Paxillin [28] providing further links between this region of Piccolo and the Actin cytoskeleton.

Similar to other Formins, Daam1 is inherently auto-inhibited by an intra-molecular interaction between its N and C terminal domains [35]. Binding of Dishevelled to its C-terminus disrupts the auto-inhibitory interaction and shifts Daam1 to an “open” conformation [32, 35]. Removal of the N-terminus of Daam1 mimics activation and leads to constitutively open conformation of the protein [32, 35]. In its “open” conformation Daam1 facilitates head-to-head dimerization, binding of Profilin, and the polymerization of ATP/G-actin into F-actin [32, 35].

**Fig 4:**
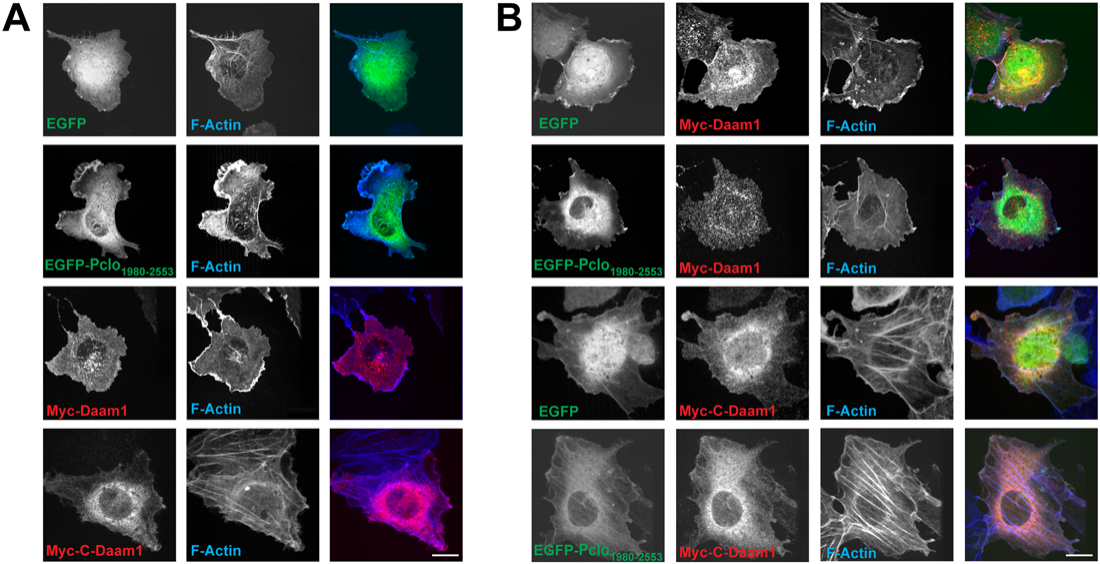
Pclo1980-2553 localizes to stress fibers induced by activated Daam1. Expression and immunostaining of COS7 cells transfected Myc tagged Daam1 isoforms (red) alone **(*A*)** or co-expresssed **(*B*)** with EGFP or EGFP-Pclo_1980-2553_ (green). Alexa Fluor-conjugated phalloidin (blue) identifies actin rich structures including stress fibers. The scale bars are both 5 μm and apply to all images in the respective panels.

To understand better the potential implications of the Daam1-Piccolo interaction, we examined whether Pclo_1980-2553_ becomes associated with higher order F-actin structures, *e*.*g*. lamellapodia or stress-fibers when expressed alone, or together with full-length Daam1 or the constitutively active C-Daam1. When transfected alone in COS7 cells both EGFP-Pclo_1980-2553_ and full-length Myc-tagged Daam1 (Myc-Daam1) exhibited a diffuse cytoplasmic pattern, but with a fraction of EGFP-Pclo_1980-2553_ associated with the phalloidin positive F-actin rich lamella (Fig 4A). Consistent with constitutive activation of Daam1 through removal of the N-terminal GBD, Myc-C-Daam1 expression led to alteration in cell shape and formation of stress fibers in nearly all cells (Fig 4A). With co-expression of EGFP-Pclo_1980-2553_ and Myc-Daam1 there was no obvious alteration in cell shape or increase in formation of stress fibers (Fig 4B). However, when EGFP-Pclo_1980-2553_ was co-expressed with Myc-C-Daam1, the phenotype was similar to that for cells expressing Myc-C-Daam1 alone (Fig 4B). Interestingly, there appeared to be some colocalization of EGFP-Pclo_1980-2553_ with phalloidin labeled stress fibers in these cells as well as some colocalization of EGFP-Pclo_1980-2553_ and Myc-C-Daam1, particularly in filopodia where there was strong labeling with phalloidin (Fig 4B). Because of the diffuse expression patterns for both EGFP-Pclo_1980-2553_ and Myc-C-Daam1, it was not possible to quantify the effects of Daam1 on Piccolo localization and *vice-versa*. Nonetheless, these findings suggest that Pclo_1980-2553_ cannot activate Daam1, but can bind to the protein once it has been activated.

**Fig 5:**
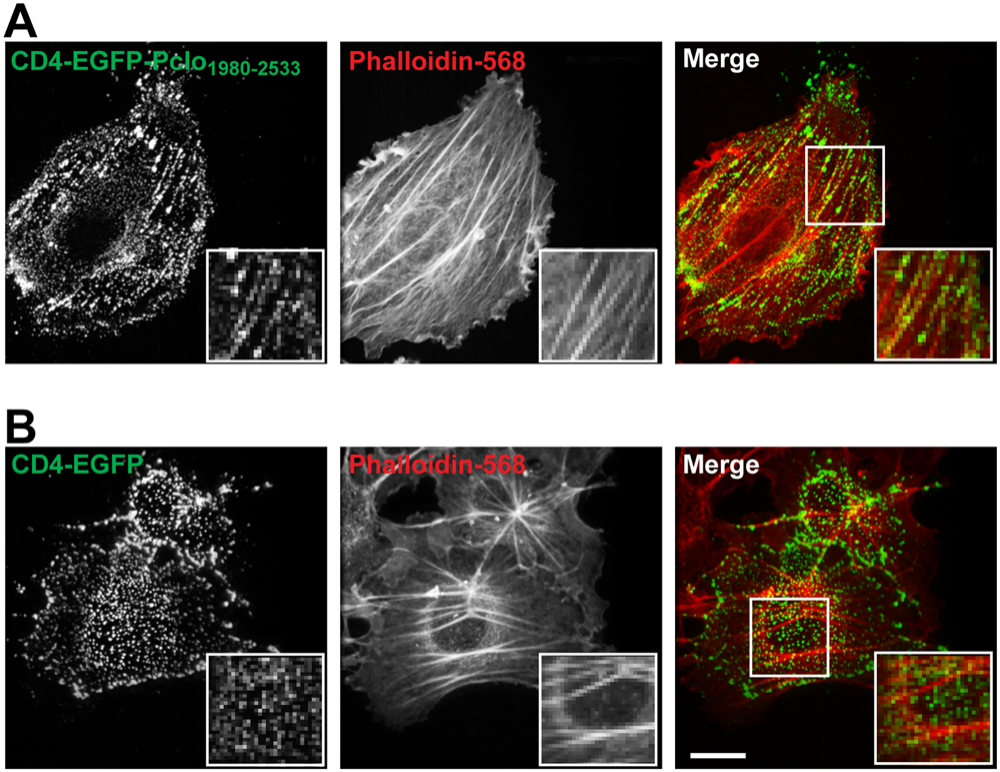
Plasma membrane targeting of Pclo1980-2553 accentuates association with stress fibers induced by activated Daam1. (***A*)** Single plane confocal images of COS7 cells expressing CD4-EGFP-Pclo_1980-2553_ chimera (green) and Myc-C-Daam1 (not displayed), treated with anti-CD4 antibodies to patch surface CD4 fusion proteins, and then, labeled with Alexa-Fluor 568-conjugated phalloidin (red). **(*B*)** As in (*A*), but with CD4-EGFP instead of CD4-EGFP-Pclo_1980-2553_ chimera. Note the presence of stress-fibers in A and B is indicative of Myc-C-Daam1 expression (see Fig 4).

To eliminate the confound of the soluble pool of Pclo_2197-2350_, we fused this fragment of Piccolo to the cytoplasmic domain of Cluster Differentiating molecule 4 (CD4) to selectively target the recombinant protein to the plasma membrane [43]. Addition of anti-CD4 antibodies to live cells expressing CD4-EGFP or CD4-EGFP-Pclo_1980-2553_ created patches of the proteins at the plasma membrane. In the vast majority (83.7 +/- 14.3%) of cells transfected with CD4-EGFP-Pclo_1980-2553_ and Myc-C-Daam1, EGFP positive puncta were found to decorate F-actin bundles traversing these cells (Fig 5A), while in no cells transfected with CD4-EGFP and Myc-C-Daam1 were EGFP positive puncta found to decorate F-actin bundles (Fig 5B). These data suggest that expression of activated Daam1 influences the distribution of Pclo_1980-2553_ by drawing it into F-actin rich structures. Because of the diffuse expression pattern of the Myc-C-Daam1, it was again difficult to assess the extent of colocalization of the Piccolo fragment and activated Daam1.

### Pclo_1980-2553_ can serve as a platform for Daam1 dependent F-actin assembly

**Fig 6:**
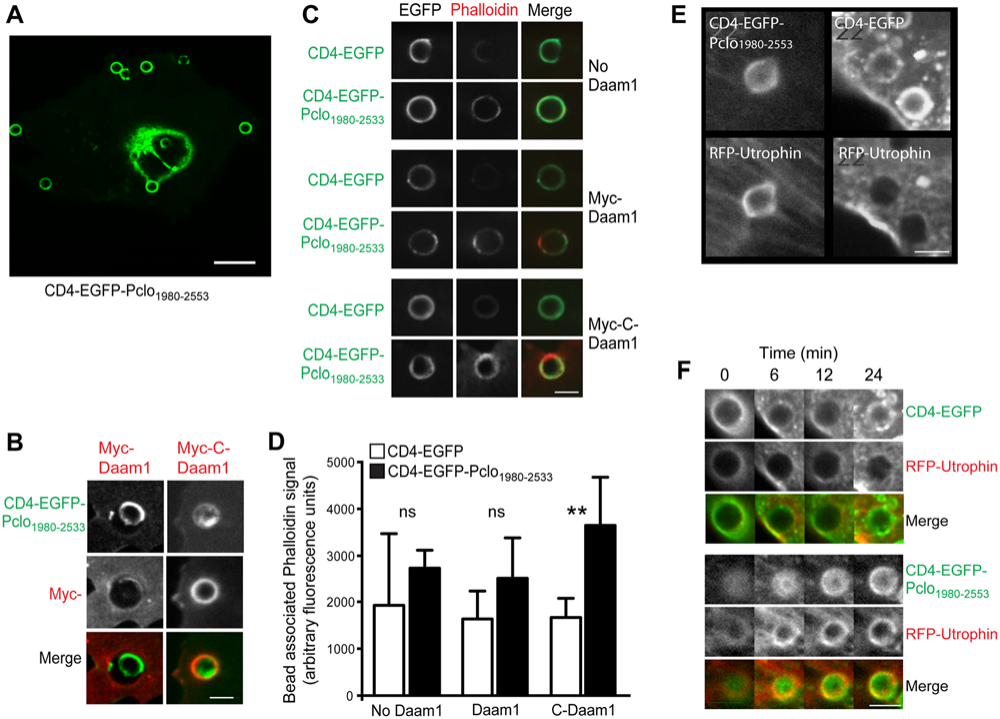
Pclo1980-2553 serves as a platform for Daam1 dependent F-actin assembly. (***A*)** Discrete round structures demonstrate that CD4-EGFP-Pclo_1980-2553_ accumulates around CD4 antibody-coupled Protein-A beads dropped onto COS7 cells. Scale bar is 20 μm. (***B*)** When coexpressed with CD4-EGFP-Pclo_1980-2553_ (top panels, green in merge) Myc-C-Daam1 (right middle panel, red in merge), but not full length Myc-Daam1 (left middle panel, red in merge), is recruited to CD4 antibody-coupled Protein A bead foci. (***C***) Visualization of F-actin accumulation by AlexaFluor 568-conjugated Phalloidin (Red) around CD4 antibody-coupled beads dropped onto COS7 cells expressing CD4-EGFP or CD4-EGFP-Pclo_1980-2553_ alone (top panels), CD4-EGFP or CD4-EGFP-Pclo_1980-2553_ with Myc-Daam1 (Middle panels) or CD4-EGFP or CD4-EGF-Pclo_1980-2553_ with Myc-C-Daam1 (bottom panels). (***D*)** Quantitation of relative accumulation of phalloidin intensity around CD4 antibody-coupled beads in COS7 cells expressing the indicated constructs. Data are expressed as mean with error bars representing standard deviation. For statistical analysis, within each group (no Daam1, Daam1, and C-Daam1) a comparison was made between cells transfected with CD4-EGFP and those transfected with CD4-EGFP-Pclo_1980-2553_ using a two-tailed t-test (** p<0.01). (***E*)** Co-expression of mRFP-Utrophin_CHD_ allows visualization of active F-actin assembly (bottom panels, red in merge) around CD4 antibody-coupled beads in COS7 cells expressing Myc-C-Daam1 and CD4-EGFP-Pclo_1980-2553_ (top left), but not CD4-EGFP (top right). **(*F*)** Time lapse image sequence of COS7 cells expressing Myc-C-Daam1 (not displayed), mRFP-Utrophin_CHD_ (Red) and CD4-EGFP (top panels) or CD4-EGFP-Pclo_1980-2553_ (bottom panels) treated with 5 μM Latrunculin-A 5 min prior to the addition of CD4-antibody coated beads. Continued accumulation of mRFP-Utrophin_CHD_ at beads on cells expressing Myc-C-Daam1 and CD4-EGFP-Pclo_1980-2553_ in the presence of 5 μM Latrunculin-A is indicative of the potent induction of F-actin assembly by these molecules. Scales bars in (*B*), (*C*), (*E*), and (*F*) are 5 μm.

Given that Piccolo is a tightly anchored component of the presynaptic AZ [40, 44, 45], we considered that within AZs, Piccolo may function as a platform/scaffold facilitating the spatial and activity-dependent assembly of F-actin within presynaptic boutons through a regulated association with Daam1. To explore this, we developed a cellular assay to examine directly whether the Pclo_1980-2553_ can act as a site of F-actin nucleation in a Daam1 dependent manner. We specifically expressed CD4-EGFP or CD4-EGFP-Pclo_1980-2553_ in COS7 cells and dropped Protein-A beads coated with antibodies against the extracellular domain of CD4 onto to the cells. We found that beads coated with CD4 antibodies, but not a non-specific IgG (data not shown), induced the recruitment/clustering of CD4-EGFP or CD4-EGFP-Pclo_1980-2553_ to the beads (Fig 6A). We then evaluated whether Myc-Daam1 or Myc-C-Daam1 were also recruited to these beads. While Myc-Daam1 was only weakly detected at CD4-EGFP or CD4-EGFP-Pclo_1980-2553_ bound beads, Myc-C-Daam1 readily accumulated around beads dropped on to cells expressing CD4-EGFP-Pclo_1980-2553_ (Fig 6B), suggesting that activation of Daam1 facilitates its recruitment to and association with Piccolo. The requirement for Daam1 to be activated in order to interact with Pclo_1980-2553_ in this assay differs from our results with the pull down assay where both full-length Myc-Daam1 and activated Myc-C-Daam1 interacted with Pclo_1980-2553_. This discrepancy may reflect inherent differences in the sensitivity of the two techniques to identify low affinity interactions or may suggest that within cells the association between Piccolo and Daam1 is subject to complex regulation.

To explore whether this arrangement also promoted the local assembly of F-actin, cells were fixed and stained with Rhodamine-Phalloidin (Fig 6C). Interestingly, we observed prominent Phalloidin labeling at CD4-EGFP-Pclo_1980-2553_/Myc-C-Daam1 bead clusters, while nominal bead associated Phalloidin labeling was seen in cells co-transfected with CD4-EGFP-Pclo_1980-2553_ and Myc-Daam1 or cells transfected with CD4-EGFP or CD4-EGFP-Pclo_1980-2553_ alone (Fig 6C and 6D).

To identify more precisely the component of Phalloidin labeling that is dependent upon Piccolo and Daam1, we established an imaging assay based on the acute addition of beads to live cells. To monitor F-actin in these experiments we expressed the F-actin binding domain (calponin homology domain) from Utrophin [46] tagged with RFP. Here, we observed low levels of RFP-Utrophin_CHD_ around CD4-EGFP or CD4-EGFP-Pclo_1980-2553_ positive beads when expressed with Myc-C-Daam1 (Fig 6E). While there appeared to be higher levels of RFP-Utrophin_CHD_ around beads in cells expressing CD4-EGFP-Pclo_1980-2553_, we sought to increase the specificity of the assay. To achieve this we acutely treated cells with a low concentration of Latrunculin A (5μM) to attenuate the assembly of F-actin. This dramatically abolished the accumulation of RFP-Utrophin_CHD_ near beads dropped onto CD4-EGFP/Myc-C-Daam1 transfected cells, while prominent labeling remained at CD4-EGFP-Pclo_1980-2553_/Myc-C-Daam1 bead clusters (Fig 6F). This is consistent with a higher kinetic assembly rate of F-actin surrounding CD4-EGFP-Pclo_1980-2553_/Myc-C-Daam1 beads overcoming the partial inhibition by the low concentration of Latrunculin-A. These results suggest that Pclo_1980-2553_ can serve as a platform for activated Daam1 mediated F-actin assembly.

**Fig 7:**
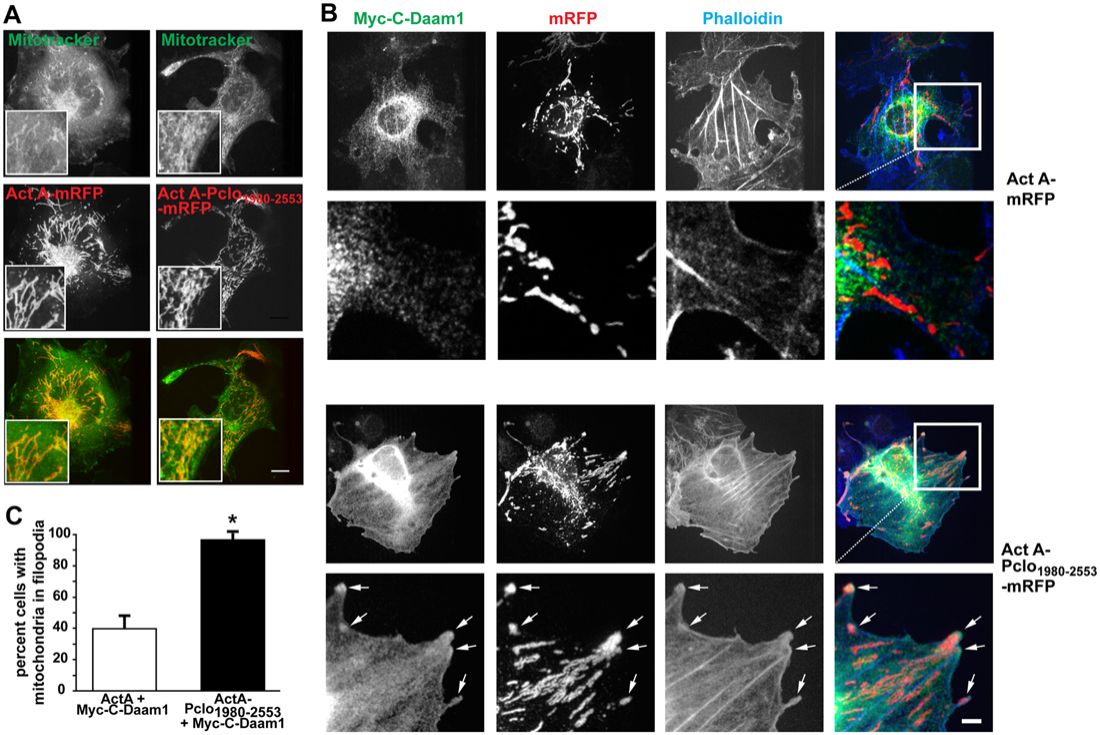
Pclo1980-2553 is targeted into filopodia when co-expressed with activated Daam1. (***A*)** Labeling of mitochondria with Mitotracker Green (upper panels, green in merge) in COS7 cells expressing ActA-mRFP or ActA-Pclo_1980-2553_-mRFP (middle panels, red in merge) demonstrates localization of Pclo_1980-2553_ sequences to the surface of mitochondria. **(*B*)** Expression of ActA-mRFP (top set of panels, red in merge) or ActA-Pclo_1980-2553_-mRFP (bottom set of panels, red in merge) with Myc-C-Daam1 (green in merge) along with Actin labeling with Alexa fluor-coupled Phalloidin (blue in merge) reveals the accumulation of ActA-Pclo_1980-2553_-mRFP labeled mitochondria in phalloidin positive filopodia. **(*C*)** Analysis of mitochondrial expression pattern indicates nearly all cells expressing ActA-Pclo_1980-2553_-mRFP demonstrate accumulation in filopodia while this pattern is seen in less than half of the cells expressing ActA-mRFP. Data are expressed as mean with error bars representing standard deviation. For statistical analysis, comparison was made between the control cells (those expressing Mcy-C-Daam1 with ActA) and cells expressing Mcy-C-Daam1 with ActA coupled to Pclo_1980-2553_ using a two-tailed t-test (* p<0.05).

To investigate further the physiological implications of the interaction between Pclo_1980-2553_ and Daam1, we created a fusion protein with the mitochondrial targeting domain (residues 438–637) of ActA from *Listeria* [47] to localize mRFP-tagged Pclo_2197-2350_ to the surface of mitochondria. As expected, adding the mitochondrial-targeting domain of ActA induced strong co-localization of the fusion protein (mRFP-Pclo_2197-2350_-ActA) with Mitotracker Green (Molecular Probes), a fluorescent mitochondrial stain (Fig 7A). Surprisingly, neither Myc-Daam1 (data not shown) nor Myc-C-Daam1 colocalized with mitochondrial-associated mRFP-Pclo_1980-2553_-ActA in the cytoplasm of co-transfected cells (Fig 7B). However, in Myc-C-Daam1 expressing cells, some of the mRFP-Pclo_1980-2553_-ActA positive mitochondria were observed along the F-actin stress fibers extending into filopodia (Fig 7B). Of particular note, the Myc-C-Daam1 immunopositive puncta nicely colocalized with mRFP-Pclo_1980-2553_-ActA within these protrusions (Fig 7B). The accumulation of mitochondria into Phalloidin/C-Daam1 positive filopodia was significantly more frequent with expression of mRFP-Pclo_1980-2553_-ActA compared to mRFP-ActA (Fig 7C), and was not observed in cells expressing Myc-Daam1 with mRFP-ActA or mRFP-Pclo_1980-2553_-ActA (data not shown). These data suggest that interactions between Piccolo and activated Daam1 are enhanced by the micro-environment within filopodia, where key regulatory proteins such as Profilin could interact with pools of PIP2 [48].

### Functional relevance of presynaptic Daam1

**Fig 8:**
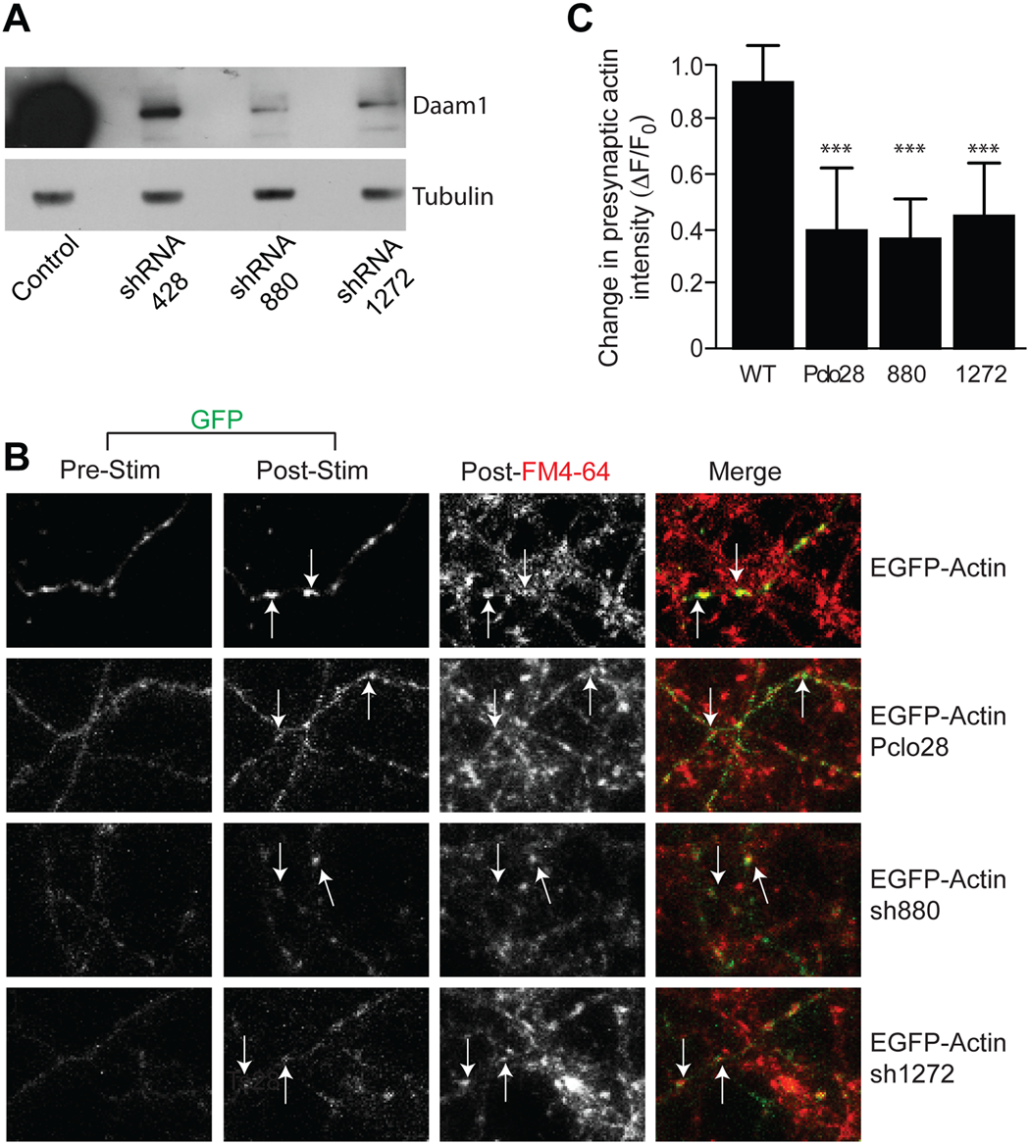
Daam1 is required for activity dependent assembly of presynaptic F-actin. (***A***) Western blot analysis of extracts (5 μg total protein) from cultured hippocampal neurons (DIV15) infected with lentiviral vectors expressing EGFP-Actin alone (control) or with the indicated shRNA targeting Daam1 (sh428, sh880, or sh1272). Antibodies against Daam1 confirm that the shRNAs markedly reduce expression of the protein relative to a control protein (tubulin). Analysis of neomycin expression from the plasmid confirms similar levels of transfection efficiencies. **(*B*)** Live cell fluorescent images of cultured hippocampal neurons (14DIV), infected with lentiviral vectors expressing EGFP-Actin (green) alone or with shRNA against Piccolo (Pclo28) or Daam1 (sh880 or sh1272). Cells were stimulated with 90mM KCl for 60 sec in the presence of FM4-64 (red) to promote the assembly of presynaptic F-actin and detect presynaptic sites capable of recycling their synaptic vesicles pools. EGFP-Actin expressed alone (top panels) readily accumulates at FM4-64 dye uptake sites, while only modest accumulation is observed in neurons lacking Piccolo (Pclo28 labeled panels) or Daam1 (sh880, sh1272 labeled panels). Arrows indicate examples of co-localization of EGFP-Actin and FM4-64 positive puncta post stimulation. **(*C*)** Quantitation of the change in EGFP-Actin fluorescence at the presynaptic boutons measured pre-and post stimulation at the FM dye uptake sites in presence of Piccolo (TS28) or Daam1 (sh1272, sh880) shRNAs demonstrates a marked decrease in Actin clustering with stimulation when either protein is targeted. Data are expressed as mean with error bars representing standard deviation. For statistical analysis, comparison was made between the control and the specific shRNA samples using a two-tailed t-test (*** p<0.001).

Our recent work has shown that Piccolo is required for the activity-dependent assembly of presynaptic F-actin, a role that is critical for the dynamic recruitment of key plasticity molecules such as CaMKII, the dispersion/recovery kinetics of Synapsin1a, and the regulated release of neurotransmitter [18]. Since Daam1 is capable of binding Piccolo in an activated state, presynaptic Daam1 could mediate some facets of Piccolo-dependent presynaptic F-actin assembly. To explore this possibility, we generated several short-hairpin RNAs (shRNAs) against Daam1 and identified three (sh428, sh880, and sh1272) that reduced Daam1 expression by >80%. We generated lentivirus vectors to express these shRNAs along with EGFP-tagged βActin (EGFP-Actin) and tested their effect on expression of endogenous Daam1 in cultured hippocampal neurons (Fig 8A). In order to determine the effect of loss of Daam1 on presynaptic Actin we monitored the dynamic behavior of co-expressed EGFP-Actin in cultured hippocampal neurons expressing two of these shRNAs (sh880 and sh1272).

To monitor changes in the intensity of EGFP-Actin specifically at presynaptic sites, we used FM4-64 to label presynaptic boutons. As previously described [18], we found that a brief addition of high K^+^ (Tyrodes with 90 mM KCl applied for 30–45 sec) caused a robust recruitment of EGFP-Actin to presynaptic boutons in wild-type neurons (Fig 8B) with about ~60% of EGFP-Actin puncta colocalizing with FM4-64. In comparing the change in intensity at FM4-64 positive sites, pre and post stimulation, we found that in neurons expressing an shRNA to knockdown Piccolo (Pclo28) the change in intensity of EGFP-Actin clusters was reduced by ~60% (Fig 8C) as previously described [18]. A similar decrease in activity induced enhancement in EGFP-Actin cluster intensity was also seen in neurons expressing either of the two shRNAs against Daam1 (Fig 8B and 8C). These data suggest that, similar to Piccolo and Profilin2 [18], Daam1 contributes to activity-dependent assembly of presynaptic F-Actin.

## Discussion

Dynamic assembly of F-actin has an established role in release of neurotransmitter, synaptic vesicle pool size, and the activity dependent recycling of SVs [7–9, 49–51], but the molecular mechanisms guiding temporal and spatial patterns of dynamic assembly of F-actin at the presynaptic bouton have been unclear. In this study, we provide evidence that Piccolo binds Daam1, a processive Formin, and that this interaction can lead to localized dynamic assembly of F-actin. Coupled with previous findings demonstrating interactions between Piccolo and other proteins involved in Actin regulation including Profilin2, GIT1, Epac2, and Abp1[12, 18, 28–30], this work supports a model in which a molecular complex centered around Piccolo directs the formation of Actin filaments from the active zone and provides a mechanisms for regulating synaptic transmission through Actin dynamics.

### Implications of Piccolo-Daam1 interactions in the presynaptic active zone

By providing cells with mechanical support and driving forces for movement, Actin is crucial for several key processes in the developing and mature nervous system including axon guidance, synapse formation, synaptic remodeling, and synaptic vesicle endocytosis [7, 10, 45]. These complex activities depend on interactions of Actin monomers and filaments with numerous other proteins. As a member of the family of processive Formins, Daam1 is part of a class of proteins that with Profilin assemble unbranched Actin filaments through hundreds of rounds of subunit addition [38, 39]. Formins have been implicated in the generation of structures that contain bundled actin filaments including stress fibers, cytokinetic rings, stereocilia, sarcomeres, and filopodia [52].

A key characteristic of Daam1 and other Formins is that they are highly regulated. Auto-inhibition of Daam1 effectively limits where and when Daam1 catalyzes Actin polymerization [37, 53]. The binding of the PDZ domain of Dishevelled to the C-terminus of Daam1 relieves the auto-inhibition and is thought to be the primary mechanism for activating Daam1 mediated Actin polymerization. Because Dishevelled is part of the non-canonical Wnt signaling pathway, Daam1 links extracellular signaling to dynamic Actin assembly [54]. Our mapping data indicate that Piccolo also binds the C-terminus of Daam1, but findings from our cellular assays suggest that within cells this interaction only occurs once Daam1 is activated. If this is the case, what role might the Piccolo-Daam1 interaction play in regulating dynamic actin assembly? A simple prediction is that the Piccolo spatially regulates Actin polymerization by interacting with activated Daam1. Indeed, the robust Actin polymerization around the CD4 antibody coated beads dropped onto cells expressing CD4-Pclo_1980-2553_-EGFP and the activated C-Daam1 (Fig 6F) demonstrates that Piccolo can localize Actin polymerization activity of Daam1. Within the intact nervous system, the restricted expression of Piccolo to axonal growth cones and presynaptic active zones [44] is predicted to cause the accumulation of activated Daam1 in these structures and thus local dynamic assembly of F-actin.

Actin polymerization by Formins such as Daam1 leads to the formation of linear as opposed to branched networks of F-Actin [41]. Ultrastructural studies have shown that presynaptic boutons contain both linear and branched networks of F-Actin filaments [10, 13, 24]. Linear F-actin networks are thought to function in the translocation of SVs from the RP to the RRP situated close to the active zone, while branched F-actin networks have been implicated in bulk endocytosis of synaptic vesicle proteins after fusion. Given the exclusive localization of Piccolo at the active zone, and the predicted plus-end orientation of F-Actin filaments emanating from the active zone, the association of Daam1 with Piccolo could facilitate the translocation of SVs via myosin motors [55, 56] toward their ultimate sites of docking and fusion.

In addition to its direct role in Actin polymerization, Daam1 also independently activates RhoA GTPase activity [35]. This suggests that a local pool of activated RhoA may result from Piccolo-Daam1 interactions. Activated RhoA interacts with effector proteins including protein kinases and actin-binding proteins, which directly and indirectly influence local assembly and disassembly of F-Actin [54, 57]. Interestingly, several studies have shown a role for non-canonical Wnt signaling in the maturation and morphogenesis of synapses, as well as in synaptic transmission [58, 59], suggesting an important role for this system in sculpting synapses. Mechanistically, it remains unclear what lies downstream of Wnt/Frizzled/Dishevelled, but Piccolo, through its interactions with Daam1, may impose a physiologically relevant spatial restriction on Daam1 dependent non-canonical Wnt signaling. Intriguingly, components of the Wnt/Frizzled/Dishevelled signaling complex are present both pre- and postsynaptically at vertebrate synapses and Daam1 has been implicated in regulation of both axonal and dendritic growth as well as lateral asymmetry in the vertebrate brain [60]. Localization of the Wnt signaling cascade via Piccolo anchoring of Daam1 may, therefore, be important for coordinating pre and post-synaptic Wnt signals.

### Piccolo as a multi-faceted regulator of the presynaptic cytoskeleton

In addition to Daam1, Piccolo has other binding partners that are involved in Actin assembly. For example, the region of Piccolo that we have shown interacts with Daam1 also interacts with Profilin and GIT1 [28, 29]. These interactions suggest that this region of Piccolo may be part of a multimeric complex. Profilins are small (12–16kD) proteins that bind monomeric actin and catalyze the exchange of ADP for ATP within globular (G) Actin to accelerate the Formin mediated polymerization of Actin monomers into filaments [61]. Of the four Profilin genes, Profilin2 is brain specific and localized to presynaptic boutons [17, 62]. In addition to binding to Piccolo, Profilin2 also binds Synapsin, Dynamin1, the Arp2/3 and WAVE (Wiskott—Aldrich syndrome protein family verprolin-homologous protein) complexes and Formin family members including Daam1 [17, 29, 63, 64]. Piccolo-Daam1 and Piccolo-Profilin interactions may serve to bring Daam1 and Profilin in proximity to each and facilitate their interaction and Actin polymerization. The interaction between Daam1 and Profilin requires activation of Daam1. This suggests that an activated Daam1 may more strongly associate with Piccolo as part of a complex with Profilin and may explain our finding that constitutively activated C-Daam1 colocalizes with Piccolo in cells while full-length Daam1 does not. Profilins also interact with phosphatidylinositol (4,5)-bisphosphate (PIP2)[48] a situation that may help regulate the assembly of F-actin in the context of the plasma membrane [61] and may explain why Piccolo’s association with Daam1 is enhanced by their mutual proximity to the plasma membrane, as observed in heterologous cells expressing ActA- or CD4- tagged Pclo_1980-2553_ (Figs 6 & 7).

GIT1 is a GTPase-activating protein (GAP) for ADP-ribosylation factors (ARFs), small GTP-binding proteins implicated in the regulation of membrane trafficking [65]. Piccolo and GIT1 can form a larger complex of proteins that includes GIT1 interacting proteins α-Liprin, FAK, PIX1, and Paxillin [28]. The latter two regulate the assembly of the actin cytoskeleton in the context of focal adhesion site [28], a feature also shared by Daam1 suggesting a potential link to integrin mediated adhesion both in growth cones and synapses.

Other domains within Piccolo have also been shown to interact with additional proteins associated with actin assembly. Abp1 has been shown to interact with a proline rich domain in the N-terminus of Piccolo [12]. Abp1 binds to both F-actin and the GTPase Dynamin and has been implicated in linking the actin cytoskeleton to Clathrin-mediated endocytosis [66]. The cAMP-binding guanidine nucleotide exchange factor Epac2 has also been shown to bind to Piccolo through the PDZ domain in Piccolo [30]. Through interactions with Rim2, the Piccolo-Epac2 complex confers cAMP-dependence to Ca^++^-evoked vesicle exocytosis in pancreatic β-cells.

Our current and previous experiments [12, 18, 28, 29, 31, 40] demonstrate that one function of Piccolo, not shared by the structurally related active zone protein Bassoon [40], is to regulate the dynamic assembly of presynaptic F-actin. This function appears crucial both for the activity-dependent recruitment of plasticity molecules such as CaMKII, as well as for the regulation of SV exocytosis during neurotransmission [18, 31]. The ability of Piccolo to associate with multiple Actin associated proteins implies that it may spatially restrict the activities of these proteins, a concept consistent with data presented here demonstrating that Piccolo can spatially control Daam1-mediated F-actin assembly. In this regard, it will be important to elucidate how upstream signaling pathways including Wnt signaling temporally control this and other Actin regulatory mechanisms and how presynaptic F-actin assembly and disassembly are modulated to influence presynaptic function.

## Materials and Methods

### Immunoprecipitation from brain lysates

Twenty P4 Sprague Dawley rat pups (Charles River) euthanized with carbon dioxide and decapitated. The brains were removed, flash frozen, and homogenized using Teflon glass homogenizer in buffer containing 5 mM MES pH 7.0, 320 mM Sucrose, and 1 mM EDTA. Lysate was cleared of nuclei and unbroken cells by centrifugation at 1000 g for 10 min at 4°C. Supernatant was hypotonically lysed by diluting with 9 volumes of buffer containing 5 mM MES pH- 7.0 and 1 mM EDTA stirring for 30 minutes on ice followed by centrifugation at 100,000 g for 1 hr at 4°C. Resultant pellet was resuspended in buffer containing 0.3 M Sucrose, 5 mM MES pH- 7.00 and 1 mM EDTA by passing the suspension through a 26 gauge needle several times. The suspension (6 mg of protein) was layered over a 2 M/1.2 M/0.8 M/0.3 M discontinuous sucrose gradient. Peak 1, 2 and 3 (P1 (0.3/0.8), P2 (0.8/1.2), P3 (1.2/2)) were obtained by centrifugation of the gradients at 300,000 g in a swing bucket rotor. Aliquots (1mg) of protein from the P2 fraction were used for each immunoprecipitation experiment. For immunoprecipitation, the P2 fraction aliquots were solubilized in 1x PBS containing 1% Triton X-100 for 1 hr at 4°C, followed by centrifugation at 4°C, at 20,000 g to clear the lysate. A previously described polyclonal antibody against Piccolo residues 1980–2553 raised in Rabbit [44] was added to the cleared lysate and incubated for 1 hr at 4°C. A 40 μl aliquot of Protein A/G beads (Pierce) was added to the lysate and incubated for 40 min at 4°C. Beads were precipitated by centrifugation at 1000 g for 1 min, washed 4X in lysis buffer (1X PBS with 1% TritonX 100) and proteins bound to the beads were eluted in 2X Laemmli sample buffer. Precipitates were fractionated on a SDS-PAGE and subjected to silver staining. Bands of interest were identified by mass spectrometry at the Vincent Coates Foundation Mass Spectrometry Laboratory, Stanford University Mass Spectrometry (http://mass-spec.stanford.edu). A control experiment using normal Rabbit IgG (Upstate) was done in parallel.

### Plasmids and vector constructions

The Piccolo_1980-2553_ clone [44] has flanking EcoRI sites that were used to clone the sequence into the EcoRI sites of EGFP C2 (Clontech) and the mRFP ActA vector (kindly provide by W.J. Nelson Stanford University). A NotI—Piccolo_1980-2553-_Stop-XhoI fragment was generated by PCR and cloned into the CD4 construct (kindly provide by Chen Gu, Ohio State University). The EGFP coding sequence with flanking NotI sites (NotI-EGFP-NotI) was then cloned into the NotI site to obtain CD4-NotI-EGFP-NotI- Piccolo_1980-2553_-Stop-XhoI. A control plasmid CD4-NotI-EGFP-NotI-Stop-XhoI was constructed similarly.

Mouse Daam1 cDNA (Accession no. AY426535) was kindly provided by Dr. Terry Yamaguchi, NCI. All Daam1 fragments were PCR amplified from original mouse Daam1 cDNA and cloned directionally into XhoI-SmaI sites of EGFP C1, EGFP C2, or EGFP C3 vectors (all from Clontech) as required. The sequence for the EGFP tag was then excised and replaced with oligonucleotides containing the sequence for the Myc epitope tag. Please see supplementary information (S1 Table) for primer sequences.

### Cell Culture and Heterologous expression

COS7 cells (ATCC) were grown in DMEM plus 10% fetal bovine serum and penicillin/streptomycin. Transfections were conducted on confluent (90–100%) cell cultures with Lipofectamine 2000 (Invitrogen) following the instructions of the manufacturer. For COS7 cells used in imaging experiments, about 20 hr post-transfection the cells were resuspended in 1 mM EDTA in 1X PBS and plated on poly-D-Lysine coated coverslips with a 1:4 dilution. Primary hippocampal cultures were prepared using a modified Banker culture protocol, as previously described [67]. Neurons were infected with lentivirus on DIV 0, prepared as previously described [31, 67]. For transfections, neurons were electro-transfected with EGFP-Daam1 on DIV 0. About 30 μg of plasmid DNA (endotoxin free, Qiagen) was electrotransfected into 1 million DIV0, E18 neurons prior to plating onto the coverslips.

### Immunoprecipitations from COS7 cell lyastes

COS7 cells were co-transfected with plasmids to express cDNAs encoding the indicated proteins. Approximately 20 hr post transfection cells were washed once with 1X PBS and harvested in 1% Triton X-100 in 1X PBS with Roche Protease Inhibitor cocktail with a cell scraper followed by a incubation for 1hr with gentle rocking at 4°C to solubilize the proteins. Centrifugation at 20,000 g for 20 min at 4°C followed to clear the lysate. EGFP antibody (Mouse monoclonal from Roche or Rabbit polyclonal from Invitrogen) and the polyclonal antibody against Piccolo residues 1980–2553 raised in Rabbit [44] were used as indicated for immunoprecipitation along with Protein A/G (Pierce).

### Immunocytochemistry

COS7 cells and neurons were fixed for 10 min at room temperature in a buffer containing 4% paraformaldehyde, 60mM PIPES pH7.0, 25mM HEPES pH 7.0, 10mM EGTA pH 8.0, 2mM MgCl_2_ and 0.12 M Sucrose. Cells were permeabilized with 0.25% Triton X100 in 1X PBS for 2 min and blocked in the blocking solution (2% Glycine, 2% BSA, 0.2% Gelatin, 50 mM NH4Cl in 1X PBS) for 30 min at room temperature. Primary antibodies were diluted as follows in blocking solution: Daam1/Mouse monoclonal (Abcam) 1:2000, Piccolo/Rabbit polyclonal (Affinity purified) [31] 1:1000, MAP2 Chicken (Jackson labs) 1:5000, and Synaptophysin (rabbit) antibodies from Santa Cruz. Secondary antibodies conjugated with fluorescent Alexa dyes were purchased from Invitrogen and used at 1:1000 dilutions. Phalloidin conjugated with Alexa 568 or 647 was purchased from life technologies, was reconstituted in 100% methanol and was used at 1:40 dilution in blocking solution. Phalloidin and antibodies were sometimes combined in the blocking solution wherever applicable.

### CD4 antibody patching assay

A CD4 antibody patching assay was developed based on a similar assay designed by Rivera and colleagues [68]. In brief, COS7 cells expressing CD4-EGFP-Piccolo_1980-2553_ or CD4-EGFP were grown on coverslips. 1μg/mL of anti-CD4 antibody (Caltag, Life technologies, Carlsbad, CA) was added to the live cells for 1hr followed by a wash with 1X PBS for 5minutes. 1ug/ml of Goat-anti Mouse IgG Alexa 488 was then added to the cells and incubated for another 1 hr prior to washing, fixation, and permeabilization as described above.

### Drop-Bead assay

COS7 cells were co-transfected with the indicated plasmids in 60 mm culture plates. About 20 hr post-transfection the cells were resuspended in 1 mM EDTA in 1x PBS and plated on poly-D-Lysine coated coverslips with a 1:4 dilution. 5μm Protein A beads (Bangs Laboratories) were coated with anti-CD4 antibody (Invitrogen) by incubation of beads and antibody for 30 min at room temperature with gentle rocking. Beads were washed three times with DMEM to remove unbound Antibody and resuspended in 200 μl of DMEM. A 20 μl aliquot of coated beads was added to each coverslip and incubated at 37°C for 15 min. Cells were then fixed with 4% paraformaldehyde and labeled with Alexa-568 phalloidin as well as Anti-Myc antibody (Santa Cruz) prior to imaging. Images were obtained at 63x PlanApoChromat (NA 1.4) oil immersion lens (Zeiss) on a Yokogawa CSU 10 spinning disc confocal head (PerkinElmer) fitted on a Zeiss Axiovert 200M microscope equipped with a photometric Cascade 512B camera (Roper Scientific) and MetaMorph 7.6 (Molecular Devices) imaging software. For experiments with latrunculin A (5μ M) (Calbiochem), COS7 cells were preincubated for 5 min with the drugs diluted 1:1000 in Tyrodes solution (from 5 mM latrunculin stock in DMSO). Images were acquired before and after drug treatment to insure that the drugs themselves had no effect on EGFP-CD4 clustering. To measure local accumulation of Actin, a region of interest immediately surrounding the beads was generated and the average pixel intensity determined in the Phalloidin channel. Background signals was measured within the cell, but away from any beads and actin specific intensity was calculated by subtracting background intensity from the average intensity in the region of interest surrounding the bead.

### Daam1 shRNA characterization and lentiviral delivery

Several potential 19 bp short hairpin RNAs (shRNA) against rat Daam1 were selected by using online freeware siDirect v 2.0. Only the shRNAs that had the highest predicted knockdowns as well as lowest off target effects were selected for down-stream processing. Using the siRNA sequences selected shRNA oligos were generated (sequences in S2 Table) and ligated into HindIII-BglII sites of the vector pZOff in front of the U6 promoter [31]. Full length EGFP-Daam1 cDNA was cloned in the same vector in front of a CMV promoter. Individual shRNAs were screened for the extent of Daam1 knock-down by transfecting HEK 293 cells followed by observation of loss of signal for EGFP and Daam1 by western blots. Three independent shRNAs (shRNA 428, shRNA880 and shRNA 1272) were selected, excised from pZOff vector by EcoRI-AccI and ligated into FUGW vector between the EcoRI-BstBI sites [31]. The shRNAs were then excised from FUGW constructs using NdeI-PacI and cloned in a modified FUGW vector carrying EGFP-βActin driven by an Ubiquitin promoter [18]. These vectors were packaged into Lentiviruses as previously described [18]. The efficacy of knockdown of endogenous Daam1 was determined by western blot analysis of extracts (5 μg total protein) from cultured hippocampal neurons (DIV15) infected at DIV0 with lentiviral vectors expressing with the indicated shRNA targeting Daam1 (sh428, sh880, or sh1272). Extracts were separated by SDS-PAGE using 3–8% gradient Tris-acetate gels (Invitrogen), transferred to nitrocellulose membranes (GE Healthcare), incubated in blocking solution (5% nonfat dry milk, 0.05% NP-40, 150 mM NaCl, 50 mM Tris, pH 7.5), and probed with primary antibodies (Daam1/mouse monoclonal (Abcam) 1:2000, tubulin/mouse monoclonal (Sigma-Aldrich) 1:5000) and secondary horseradish peroxidase (HRP)-conjugated antibodies (GE Healthcare) in blocking solution. Protein bands were detected by HRP chemiluminescence (PerkinElmer) and exposure to film. Lentiviruses co-expressing the short hairpin RNA (shRNA) against Piccolo (Pclo28) and EGFP-Actin were used as described previously [18].

### Live Imaging

All live imaging experiments were performed on a custom-built scanning confocal microscope (Zeiss Axiovert 200M) equipped with a 40x objective (1.3 NA; Zeiss Plan Neofluar), 488–514 nm laser (Spectraphysics) and using OpenView software (written and provided by Noam Ziv, Technion Institute, Haifa, Israel). Neuronal coverslips were mounted in a custom-built chamber designed for perfusion and electrical stimulation, heated to 37°C by forced-air blower and perfused with Tyrode’s saline solution (25 mM HEPES, 119 mM NaCl, 2.5 mM KCl, 30 mM glucose, 2 mM CaCl, 2 mM MgCl_2_, 50 μM CNQX, 10 μM APV, pH 7.4).

### Hippocampal culture and lentiviral infection

Primary hippocampal cultures were prepared using a modified Banker culture protocol [69]. Briefly, timed pregnant Sprague-Dawley rats were euthanized with carbon dioxide and decapitated and embryos (E18.5) harvested. Hippocampi of embryos were removed and dissociated in 0.05% trypsin and neurons plated at a density of 165 cells/mm^2^ on poly-L-lysine coated coverslips, and inverted over a glial feeder layer 1 hr after plating as previously described [18]. Thereafter, cultures were maintained in Neurobasal medium supplemented with B27 and 2 mM GlutaMAX (Invitrogen). Neurons were infected at DIV0 with indicated lentiviral vectors prepared as previously described [18, 31, 67] and maintained in culture until used for experiments between DIV14-16.

### Actin clustering assay

DIV 14 Neurons that had been earlier infected with Lentiviral vectors carrying indicated shRNAs and EGFP-Actin were treated with Tyrode’s solution containing 90 mM KCl for 60sec along with ~1μg/ml FM4-64 dye (Invitrogen), followed by 30 sec incubation in normal Tyrodes with 1 μg/ml FM dye. Neurons were then washed in Tyrodes solution for 1–2 min before imaging as described [18]. Images of EGFP-Actin clusters before and after 90 mM KCl treatment conditions were compared to determine the relative change in F-Actin at synaptic sites identified using FM4-64 labeling as described [18].

### Quantification of EGFP-Actin cluster

EGFP-Actin fluorescence intensity at presynaptic boutons (based on colocalization with FM4-64) was measured with OpenView software as described [18]. Intensity values from each set of 3 pre-stimulation images were averaged to give *avg F*_*o*_, and those from post-stimulation to give *avg F*_*post-stim*_. These results were then expressed as change in fluorescence (*avg F*_*post-stim*_—*avg F*_*o*_,) vs. initial fluorescence (*ΔF*/*F*_*o*_), and averaged for all EGFP-Actin clusters in a field of view using Microsoft Excel. GraphPad Prism was used for graph plotting and statistical analyses. Fluorescence intensity values were averaged across experiments for a given condition (wild-type, Pclo28, shRNA 880 or shRNA 1272), converted to % increase and plotted, along with their standard deviation, for each set of conditions.

### Ethics Statement

All animal studies were approved by The Stanford University Institutional Animal Care and Use Committee.

## Supporting Information

S1 Table Sequences of oligonucleotides used for PCR generation of DNA fragments. The sequences (5’ to 3’) of the oligonucleotides used to produce DNA fragments for generation of the plasmids used to express the different recombinant proteins.
(DOCX)

S2 Table Sequences of shRNA targets. The sequences (5’ to 3’) of the targets for the indicated shRNAs.
(DOCX)
