# Supplementary material for: Piccolo Directs Activity Dependent F-Actin Assembly from Presynaptic Active Zones via Daam1
Source: PLoS One. 2015 Apr 21;10(4):e0120093. doi: 10.1371/journal.pone.0120093 (PMC4405365; doi:10.1371/journal.pone.0120093)
Supplement: S1 Table — The sequences (5’ to 3’) of the oligonucleotides used to produce DNA fragments for generation of the plasmids used to express the different recombinant proteins. (DOCX) [file pone.0120093.s001.docx]

| Daam1 XhoI C2.5 | AGGAGAGAATTCTCGAGAACCATGGCC |
| --- | --- |
| Daam1 SmaI C2.3 | TTAATTTTTAACCCGGGTTCCTTGCAT |
| Daam1 FH1-Xho C3.5 | CCCGACTCCACACCTCTCGAGAACTTTAATATCAAG |
| Daam1 FH1-SmaI C3.3 | CAAGAAGTAGAATATTCCCGGGCCCACCAATGCC |
| Daam1 FH2-XhoI-C1.5 | CCTCCTGGTGCTCGAGTGAGTCTAACACTC |
| Daam1 FH2-SmaI C1.3 | GAAGCGAAACAGTAAAATGAGCCCGGGAGAAAAAGG |
| CD4-NotI-EGFP-5’ | AATATTTAGCGGCCGCCATGGTGAGCAAGGGCGAGGAGC |
| CD4-NotI-EGFP-3’ | TATAATATGCGGCCGCCTTGTACAGCTCGTCCATGCCGAGAGTGATC |
| CD4-EGFP-Stop-XhoI-3’ | TATAATATCTCGAGTTACTTGTACAGCTCGTCCATGCCGAGAGTGATC |
| CD4-44AII-NotI-5’ | ATTATAAGCGGCCGCCACGAGGCTCACCAGGCA |
| CD4-44AII-XhoI-3’ | CATGCTGCTCGAGGTCACGAGTTTAAGGACATAG |
