# Supplementary material for: Piccolo Directs Activity Dependent F-Actin Assembly from Presynaptic Active Zones via Daam1
Source: PLoS One. 2015 Apr 21;10(4):e0120093. doi: 10.1371/journal.pone.0120093 (PMC4405365; doi:10.1371/journal.pone.0120093)
Supplement: S2 Table — The sequences (5’ to 3’) of the targets for the indicated shRNAs. (DOCX) [file pone.0120093.s002.docx]

| shRNA-428 | AAAGCCCATGAGGTTTGTAAC |
| --- | --- |
| shRNA-880 | TGTTCATTAGTGCCTTTATACAG |
| shRNA – 1272 | AGCCTGTTATAGATAAATTAAGG |
